# Anomaly-informed remaining useful life estimation (AIRULE) of bearing machinery using deep learning framework

**DOI:** 10.1016/j.mex.2024.102555

**Published:** 2024-01-05

**Authors:** Pooja Kamat, Satish Kumar, Shruti Patil, Ketan Kotecha

**Affiliations:** a Symbiosis Institute of Technology, Symbiosis International (Deemed University), Lavale, Pune, Maharashtra, India; b Symbiosis Centre for Applied Artificial Intelligence, Symbiosis International (Deemed University), Lavale, Pune, Maharashtra, India

**Keywords:** Bearings, Remaining useful life, Anomaly detection, Autoencoder: LSTM, Anomaly-Informed Remaining Useful Life Estimation (AIRULE) using Hybrid LSTM models

## Abstract

A rolling bearing is a crucial element within rotating machinery, and its smooth operation profoundly influences the overall well-being of the equipment. Consequently, analyzing its operational condition is crucial to prevent production losses or, in extreme cases, potential fatalities due to catastrophic failures. Accurate estimates of the Remaining Useful Life (RUL) of rolling bearings ensure manufacturing safety while also leading to cost savings.

• This paper proposes an intelligent deep learning-based framework for remaining useful life estimation of bearings on the basis of informed detection of anomalies.
• The paper demonstrates the setup of an experimental bearing test rig and the collection of bearing condition monitoring data such as vibration data.
• Advanced hybrid models of Encoder-Decoder LSTM demonstrate high forecasting accuracy in RUL estimation.

## Graphical abstract

**Figure:**
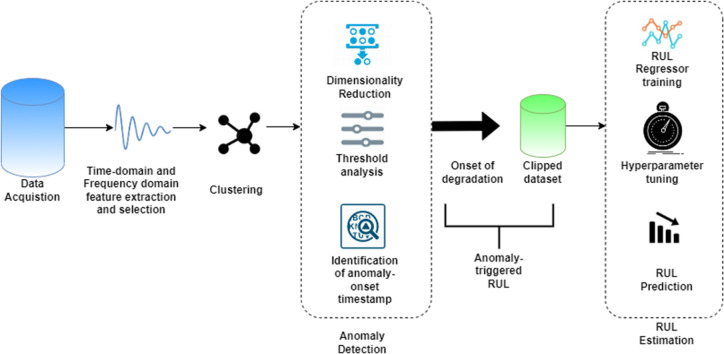

Specifications table

**Table:** 

Subject area:	Engineering
More specific subject area:	Deep Learning
Name of your method:	Anomaly-Informed Remaining Useful Life Estimation (AIRULE) using Hybrid LSTM models
Name and reference of original method:	Clustering and Change Point Detection Algorithm (CPDA) [Kundu, P., Chopra, S., & Lad, B. K. (2019). Multiple failure behaviors identification and remaining useful life prediction of ball bearings. Journal of Intelligent Manufacturing, 30, 1795–1807. DOI 10.1007/s10845–017–1357–8]
Resource availability:	Data: Data available on request Software: Google Colab, Scipy library

## Method details

**Fig. 1:**
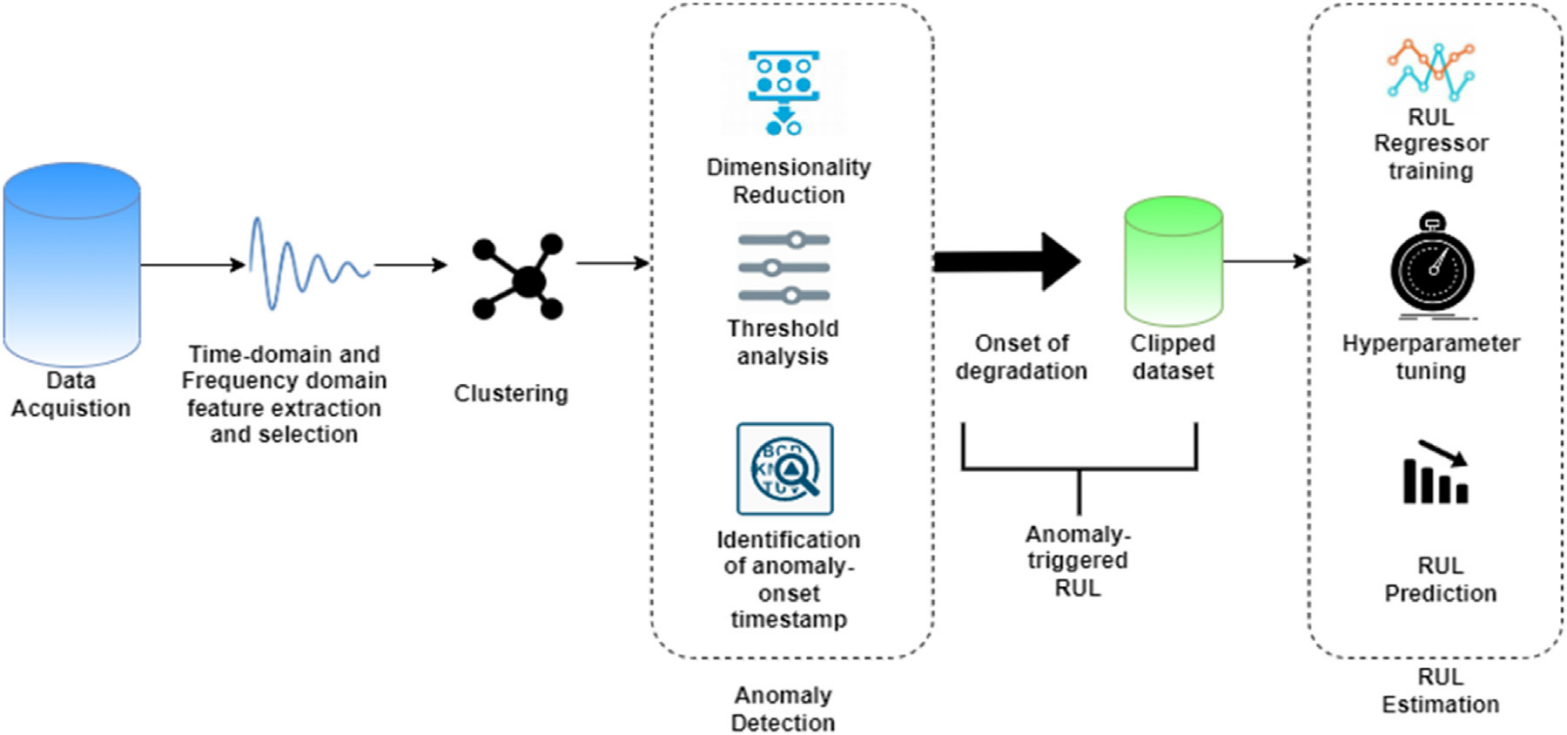
Anomaly-informed remaining useful life estimation (AIRULE) method.

In building a deep learning model for Remaining Useful Life (RUL) estimation, a significant challenge lies in determining the accurate reference truth RUL for the gathered data. As the machine approaches failure, RUL diminishes linearly [1]. However, estimating RUL beforehand is not straightforward. Many studies establish a maximum RUL threshold and assume a continuous RUL progression thereafter. Determining the optimal RUL is arbitrary, as various systems have different optimum values [2]. There is limited research on identifying anomalies and anticipating RUL from the time the abnormality arises. RUL calculations have dominated pertinent PHM research throughout the wear-out stage [3]. The detection of machinery degradation is not mentioned in most of the procedures. It's difficult to estimate the RUL while the machinery is in good shape, thus detecting the first signs of wear is a useful technique to undertake defect predicting. To have strong RUL estimations, most of these algorithms require a large quantity of data to comprehend prior examples [4]. As a result, these methodologies can only be employed to a limited extent when there isn't enough data. The crux of this research lies in devising an integrated framework termed as Anomaly-Informed Remaining Useful Life Estimation (AIRULE) ' grounded in deep learning. This system not only informs the inception of anomalies but also forecasts the remaining operational life of the machinery. To discern the most influential features, feature ranking and selection techniques are implemented [5]. Unsupervised clustering algorithms are employed for anomaly trend analysis, while supervised deep learning methods are harnessed for pinpointing anomaly information and estimating RUL. The RUL regressors are exclusively trained and fine-tuned on the clipped dataset following the anomaly's start, rather than the entire dataset [6]. Fig. 1 illustrates the methodology of the Anomaly-Informed Remaining Useful Life Estimation (AIRULE) framework. The framework can be divided in five stages as follows: (i) Dataset gathering (ii) Feature extraction and selection (iii) Clustering for pattern mining (iv) Anomaly inception detection and (v) RUL prediction.

## Method validation

### Datasets

**Fig. 2:**
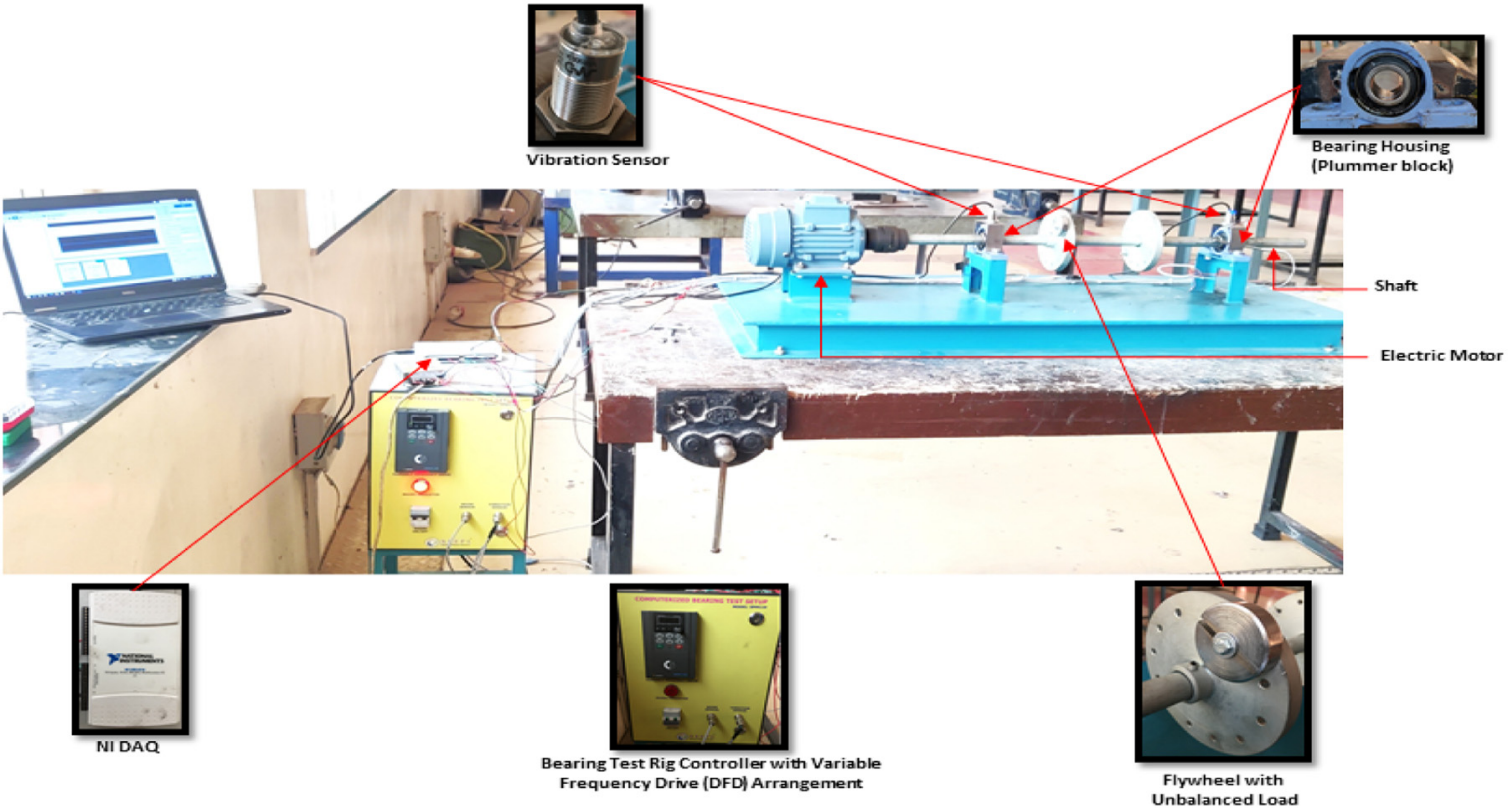
Experimental bearing test rig setup.

The step 1 involves data gathering. The experimental bearing test equipment is designed to monitor and determine the useful life of bearings. 25 mm size journal bearings were used in this study. The bearings vibrations were recorded using two accelerometer sensors mounted on. The bearings were operated at a motor speed of 1200 rpm and initially normal vibration values were recorded at a sampling frequency of 1 kHz. Artificially faults were introduced in the bearings by applying a radial load of 100 N and the bearings were operated till failure by running continuously for a period of 11 h (approx. 39,600 s). The data log was maintained via a DAQ LabView interface. The datalog consisted of vibration values of both the accelerometer sensors at a particular timestamp. Fig. 2 shows the experimental setup snapshot of the experimental bearing test rig.

### Model parameters and performance metrics

**Fig. 3:**
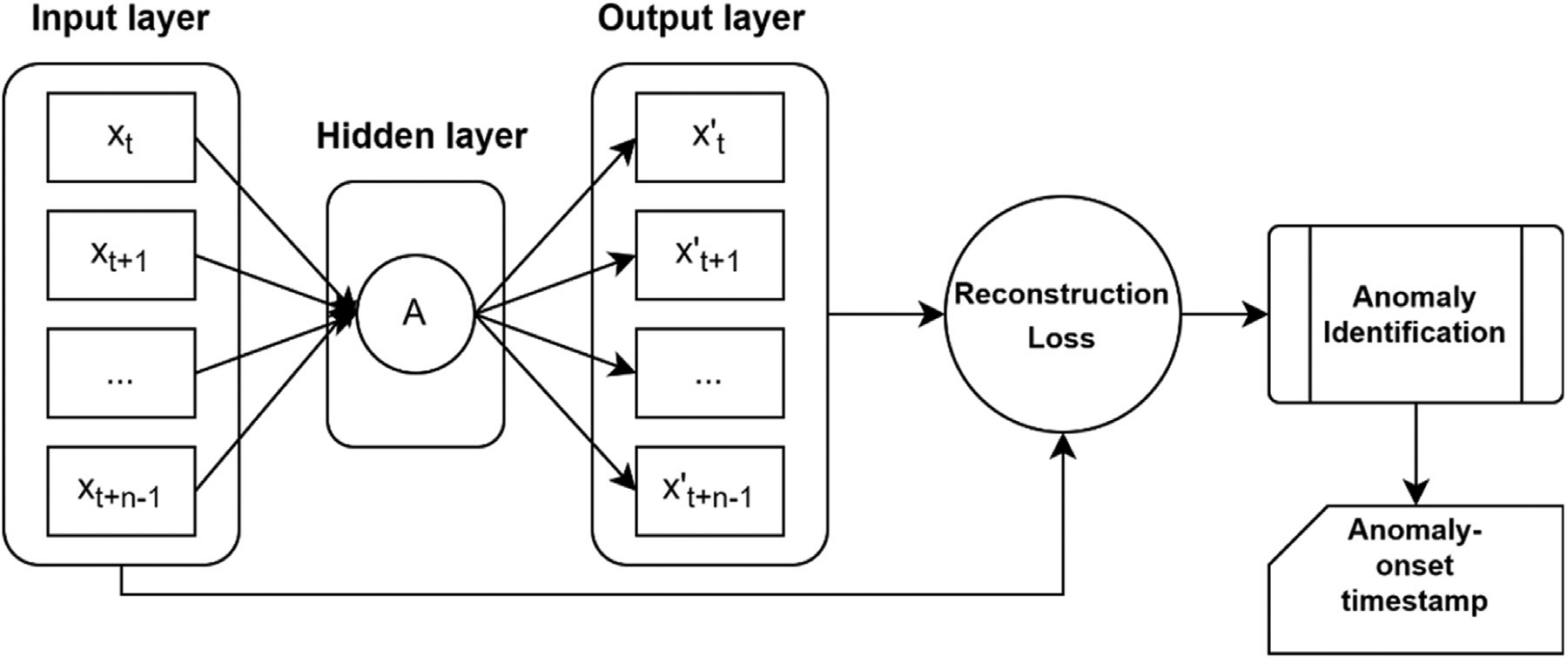
Anomaly detection using autoencoder architecture.

Step 2 involves the extraction time-domain and frequency-domain features from gathered vibration data. These features contribute to the machine condition monitoring analytics. Feature selection strategies are applied to select most desirable features. These features can then be sent to the third step for cluster analysis. Normal and anomalous clusters can be identified in the clustering stage. Anomaly detection is the process of identifying unusual occurrences that stand out from the rest of the data. Outliers or atypical samples are more intriguing than normal samples in a variety of applications [7]. Anomaly identification is the first and most important step in any predictive maintenance endeavour. Machine health monitoring can be aided by anomaly detection [8]. Identifying the initial abnormality's timestamp may offer further information about the machinery's remaining useful life (RUL) [9]. Supervised anomaly identification techniques require awareness of the instance labels ahead of time. Outliers seldom show in the distribution of class labels, which is heavily skewed toward the normal class [10]. Unsupervised or semi-supervised algorithms come to aid in such situations wherein they treat outliers in data as anomalies after pattern mining from partially-labelled data. They scale anomaly occurrence on basis of a threshold value depending on the error difference between a known and an unknown pattern [11]. Autoencoders are semi-supervised models that detect anomalies based on reconstruction loss. Autoencoders are ideally suited for anomaly detection applications since loss has no labels. A typical Autoencoder architecture is shown in Fig. 3.

In the fourth step of the analysis, the research study deployed the hybrid AE-LSTM (Autoencoder-Long Short Term Memory) model for anomaly detection. Initially, the feature vector, encompassing selected attributes, was input into the hybrid AE-LSTM model [12]. The model underwent training using both typical and aberrant clusters. Consequently, the data was encoded into an array of LSTM features that encompassed observations, attributes, and an n-timestep window. As the sliding window traversed the sample space, the encoder model learned from the underlying patterns in the temporal data. On the decoding end, the model attempted to reproduce the original data. At each stage of this process, the mean absolute error, referred to as the reconstruction loss (MAE), was calculated. In cases involving anomalous samples, the reconstruction loss exhibited notably high values, potentially resulting in the sample being flagged as abnormal. Notably, the Autoencoder component efficiently reduced dimensionality during the reconstruction process, preserving only the crucial aspects of the vibration signals. Concurrently, the LSTM model provided insights into the progression of machinery deterioration over time [13].

The final fifth step of the Anomaly-Informed Remaining Useful Life Estimation (AIRULE) system has the primary objective to avoid RUL estimate in normal procedures when the RUL is not properly specified. This is the period when the first fault/anomalous vibration has not occurred. AIRULE learns and validates RUL estimators as soon as the beginning of degradation is detected by an anomaly detection phase or other methods [14,15]. RUL estimate is a time series problem in which the aim is to make a prediction about the RUL value corresponding to a certain time. The time-series data is a continuous data which is divided into fixed-sized segments determined by the “window-size”. Each of these segments is labelled by a linearly decreasing RUL value. Many LSTM variants such as Vanilla LSTM, Stack_LSTM, Bidirectional LSTM, CNN_LSTM, Conv_LSTM, AE-LSTM and Encoder-Decoder LSTM were used in this study for RUL estimation.

The hyper parameters used for optimization in AIRULE framework are shown in Table 1:

**Table 1:** Hyperparameters used in AIRULE framework.

Model Parameters	Type/Range/Value
activation_function	ReLU
Number_of_epochs	50
optimizer	adam
loss	mse
step_size	10
kernel_size	1
pool_size	2
number_of_filters	64

The hyperparameters were fine-tuned for the selected features to achieve highest R2 accuracy in RUL forecasting. R2 (R-squared) accuracy, commonly referred to as goodness of fit, measures how well a model matches the data. R2 is a statistical measure of how closely the regression predictions correspond with the actual data points. When R2 equals 1, the regression's predictions mirror the data precisely. R2 can be calculated as follows:

(1) $$R^{2}=1-\frac{\sum_{i=1}^{n}{\left({\hat{y}}_{i}-y_{i}\right)}^{2}}{\sum_{i=1}^{n}{\left(y_{i}-{\bar{y}}_{i}\right)}^{2}}$$

Here ${\hat{y}}_{i}$ is the predicted value, $y_{i}$ is the true value and ${\bar{y}}_{i}$ is the mean over n samples

The R2 accuracy values are represented in percentage form. The LSTM models are analyzed over higher R2 accuracy percentages for RUL prediction.

## Results and discussion

**Fig. 4:**
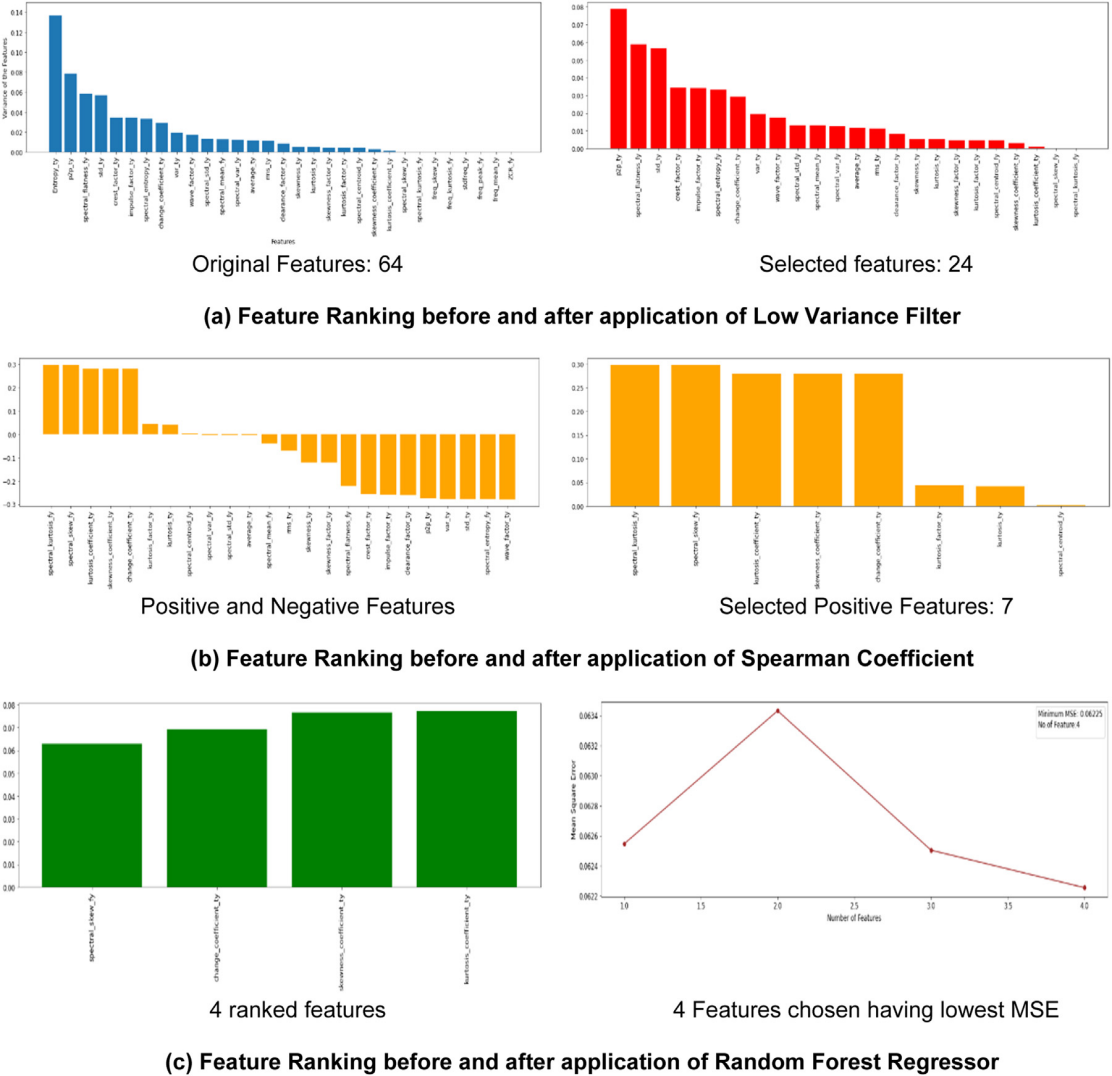
Feature selection of time and frequency domain features of experimental bearing test rig dataset.

Initially feature extraction of raw vibration data collected by both the accelerometer sensors (X and Y) are extracted. Around 20 time-domain features each and 14 frequency domain features each of X and Y are extracted. The total 64 features are further sent for feature engineering process. The 64 features were first to the feature selection techniques of Low Variance filter, Spearman Correlation Coefficient and Random Forest regressor. Fig. 4 describes the results of the feature selection technique.

**Fig. 5:**
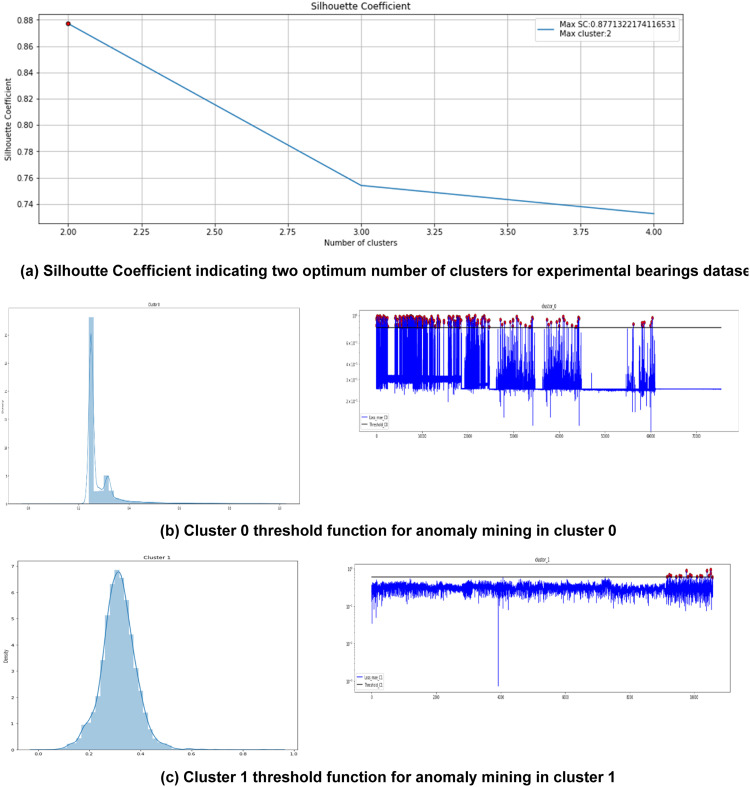
K-Means clustering for anomaly pattern mining using time and frequency domain information for experimental bearings dataset.

The selected features are then sent for step 3 of AIRULE framework clustering for anomaly pattern mining. Fig. 5 presents the Silhouette Coefficient value and clustering analysis for generation of normal and abnormal clusters of experimental bearings.

**Fig. 6:**
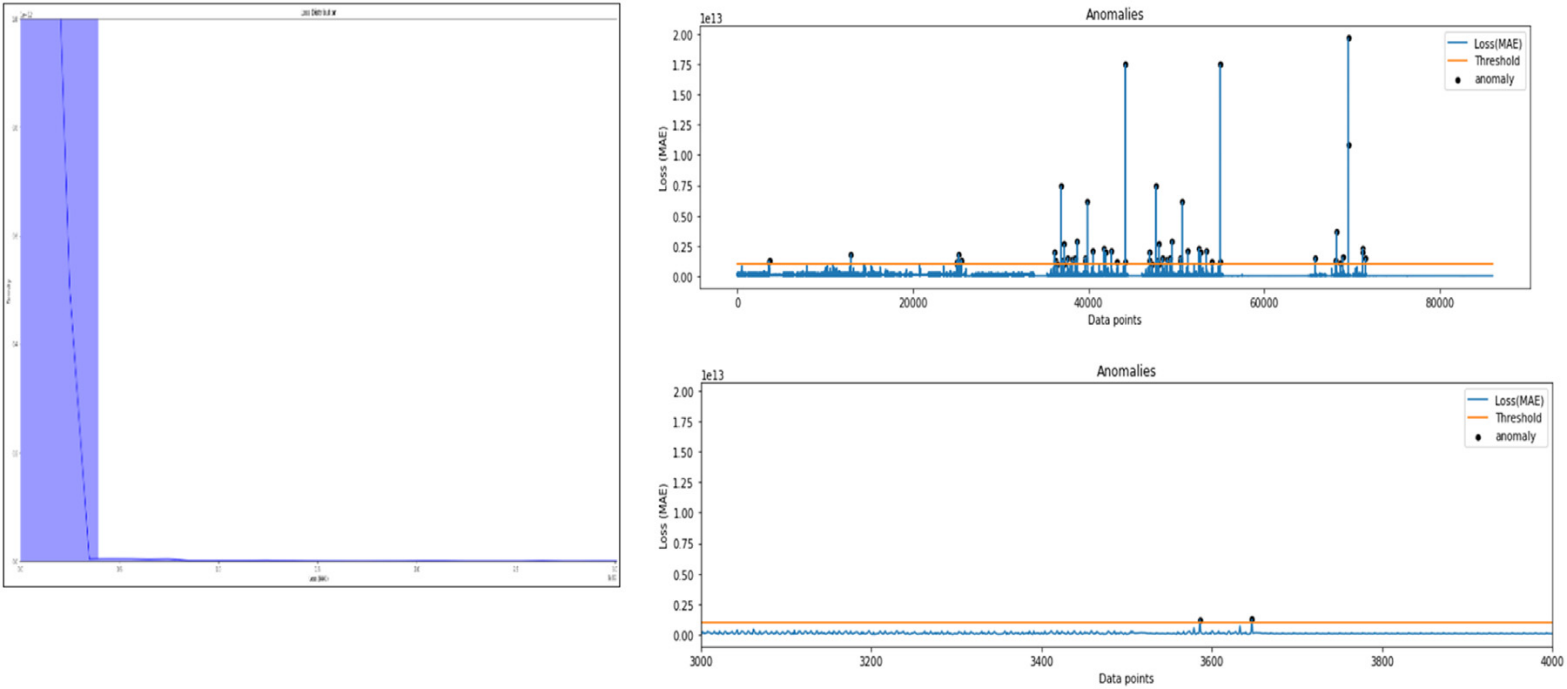
AE-LSTM reconstruction loss threshold for anomaly informed based on time and frequency domain features for experimental bearings dataset.

The next stage of anomaly detection for informing the initialisation of anomaly occurrence is applied on normal and abnormal clusters. The Autoencoder-LSTM reconstruction loss probability distribution function helps to detect the threshold for anomaly and the timestamp indicating the start of anomaly is identified. Fig. 6 depicts this process.

**Fig. 7:**
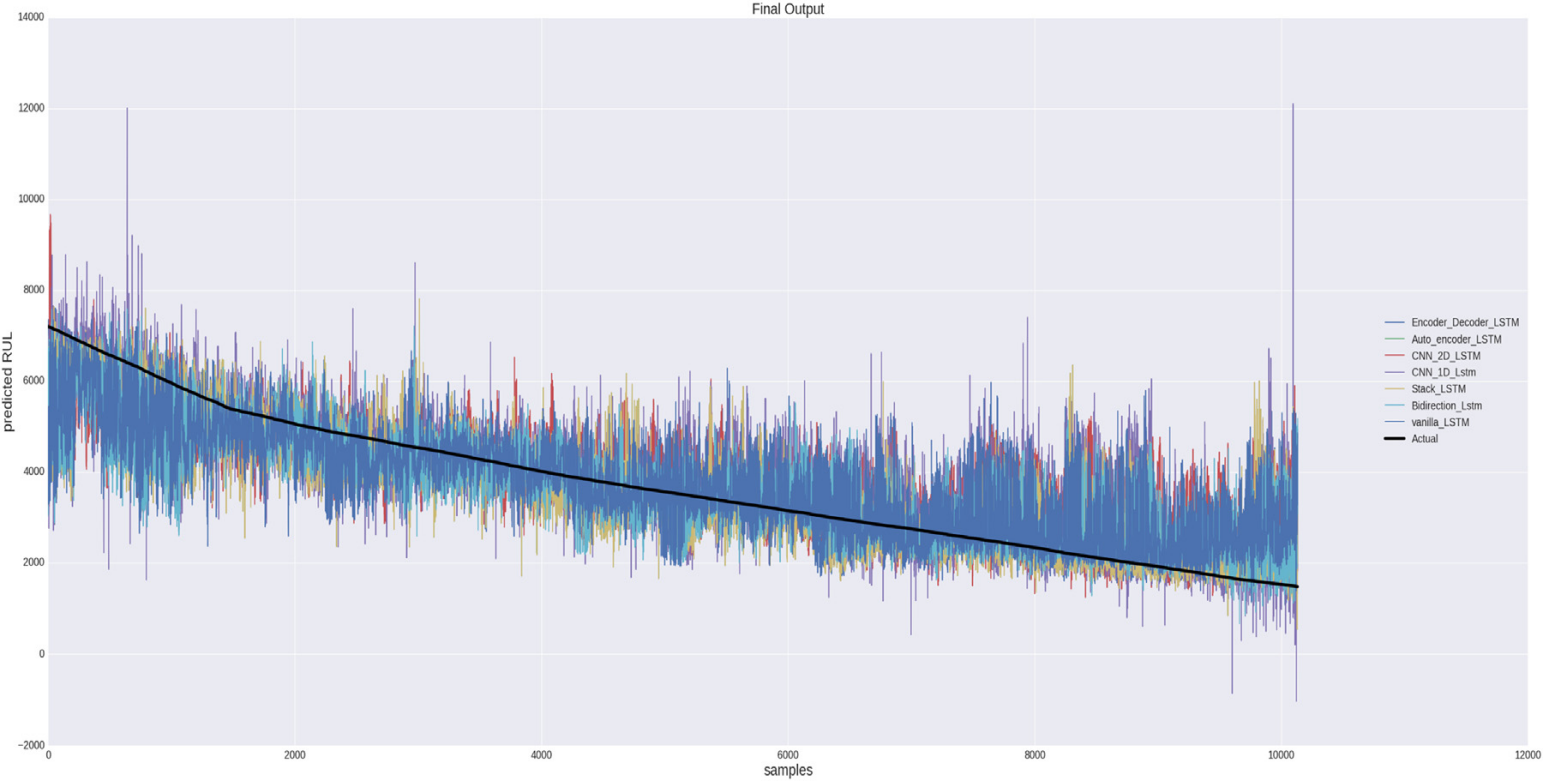
Anomaly-informed remaining useful life estimation (AIRULE) using time and frequency domain features for experimental bearings.

At the end the anomaly triggered dataset is sent for RUL estimation stage using various LSTM variants. Fig. 7 depicts the predicted findings for the test set's remaining usable life. It is clear that forecasted RUL values fall progressively over the test vibration samples.

Table 2 presents the R2 accuracy and MSE values for remaining useful life estimation by each of the LSTM variants.

**Table 2:** RUL prediction results for experimental bearings dataset using LSTM variants.

Prediction Results		Vanilla_LSTM	Bi LSTM	Stack LSTM	CNN 1D_LSTM	CNN 2D_LSTM	AE LSTM	Encode Decode LSTM
RUL Prediction	R2 Accuracy (%)	83.90	88.57	80.51	87.98	81.33	89.00	90.64
	MSE	0.01084	0.00769	0.01531	0.00768	0.01411	0.00650	0.00624

**Fig. 8:**
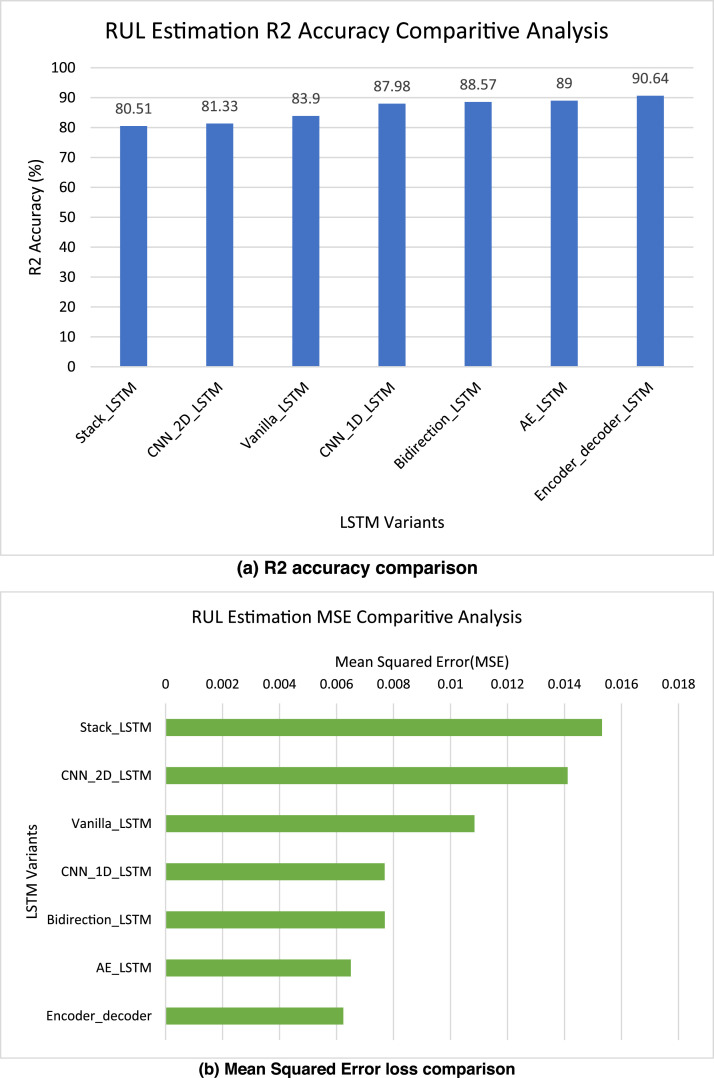
Comparative performance analysis of LSTM variants for experimental bearings RUL prediction.

The performance analysis of the LSTM variations for RUL estimation is shown in Fig. 8(a) and (b) for the experimental bearings dataset. As observed from the figures, Encoder-Decoder LSTM model achieved highest R2 accuracy and least Mean Squared Error (Loss) amongst all variants.

## Ethics statements

Not applicable

## Funding statement

This work was supported by the Research Support Fund (RSF) of Symbiosis International (Deemed University), Pune, India.

## CRediT authorship contribution statement

**Pooja Kamat:** Conceptualization, Methodology, Formal analysis, Data curation, Writing – original draft. **Satish Kumar:** Conceptualization, Methodology, Writing – review & editing, Resources, Supervision, Funding acquisition. **Shruti Patil:** Conceptualization, Resources, Supervision, Funding acquisition. **Ketan Kotecha:** Writing – review & editing, Resources, Supervision, Funding acquisition.

## Declaration of competing interest

The authors declare that they have no known competing financial interests or personal relationships that could have appeared to influence the work reported in this paper.

## Data Availability

Data will be made available on request.
